# Natural Selection as a Process That Increases Metabolic Entropy Production? A Regime-Dependent Analysis in Open Chemostat Systems with Michaelis–Menten Kinetics and Mutation–Selection Dynamics

**DOI:** 10.3390/e28090983

**Published:** 2026-09-03

**Authors:** Luca De Gioia

**Affiliations:** Department of Biology and Biotechnology, University of Milano-Bicocca, 20126 Milano, Italy; luca.degioia@unimib.it

**Keywords:** entropy production, natural selection, chemostat, Michaelis–Menten kinetics, maximum entropy production principle, mutation–selection dynamics, eco-evolutionary feedback

## Abstract

We analyse whether Darwinian natural selection acts as a metabolic-entropy-production-increasing process in a model of an open chemostat ecosystem, where Michaelis–Menten uptake kinetics are grounded in a thermodynamically consistent mesoscopic chemical-reaction-network model, and the continuum limit of the discrete replicator equation is derived as a Crow–Kimura reaction–diffusion process. On the quasi-static ecological manifold, an exact Fisher-type identity yields a monotonic increase in the metabolic entropy-production rate, proportional to the uptake-rate variance. Solving the reduced moment equations in closed form, under an explicit quasi-static and zero-skewness closure, identifies a thermodynamic ceiling imposed by mass conservation, while phenotypic trade-offs produce a finite sub-ceiling. When ecological and evolutionary timescales become comparable, a frozen-parameter Routh–Hurwitz analysis identifies an instantaneous spectral instability boundary whose scope and self-consistency are assessed for both unbounded and bounded trait models. The results of this analysis delineate the precise dynamical and biochemical conditions under which selection increases metabolic entropy production and the regimes in which that tendency is reshaped.

## 1. Introduction

The relationship between biological evolution and thermodynamics has attracted sustained theoretical interest since Schrödinger’s celebrated observation that living organisms maintain their internal order by exporting entropy to the environment [1]. In the context of Darwinian evolution, the question of whether natural selection acts merely as a biological algorithm or as a fundamental thermodynamic optimisation principle has been debated intensively over the past three decades [2,3,4].

The Maximum Entropy Production Principle (MEPP) asserts that non-equilibrium systems evolve toward states that maximise the rate of entropy production subject to the external constraints imposed on them [2,5]. Despite compelling phenomenological evidence in atmospheric and geophysical systems [6], the MEPP lacks a universally accepted derivation from first principles, and its biological applicability remains contested [7,8,9]. Concurrently, the advent of stochastic thermodynamics has provided rigorous tools to quantify entropy production at the microscopic scale of open biochemical networks [10,11,12,13].

A substantial body of earlier work has applied non-equilibrium thermodynamic reasoning to biochemical reactions, enzyme kinetics, and metabolic systems. Hill’s treatises on free-energy transduction and biochemical cycle kinetics [14,15], together with Schnakenberg’s network formulation [16], provide classical foundations for thermodynamic bookkeeping in biochemical and stochastic reaction networks. Westerhoff and van Dam [17] extended non-equilibrium thermodynamics to biological free-energy transduction and metabolic control. Qian and collaborators developed explicit non-equilibrium thermodynamic descriptions of open biochemical networks, including entropy production, free-energy dissipation, and stochastic steady states [12,18,19,20,21]. Andrieux and Gaspard derived fluctuation relations for currents using Schnakenberg network theory [22]. More recent work on chemical-reaction-network circuit theory and thermodynamically consistent coarse graining further clarifies how reaction currents, thermodynamic forces, and dissipation remain well defined and mutually consistent when detailed biochemical mechanisms are coarse-grained into simpler effective descriptions [23,24].

Complementing this literature, several studies have directly considered entropy-production extremum principles in enzyme kinetics and metabolic evolution. Dobovišek et al. [25] derived an entropy-production maximum with respect to enzyme rate constants in a reversible kinetic scheme and compared the prediction with evolved beta-lactamases. A later analysis investigated maximum entropy production and Shannon entropy in a two-step enzyme model [26], and subsequent work extended MEPP-based analysis to the self-organisation of open enzyme-catalysed reaction schemes [27]. Unrean and Srienc [28] combined a reduced metabolic-network model with adaptive-evolution experiments and reported an increase in entropy production toward a model-dependent maximum. Stucki [29] studied optimal efficiency and coupling in oxidative phosphorylation using linear non-equilibrium thermodynamics; Caplan and Essig [30] provided a broader linear non-equilibrium thermodynamics (LNET) treatment of biological energy transduction. At the systems level, Heinrich and Schuster [31] and Fell [32] pioneered the use of variational and optimisation principles—such as maximising metabolic flux or minimising enzymatic protein costs—to understand the evolutionary tuning of enzyme regulation and metabolic control. Thermodynamic constraints in metabolic-network models were subsequently incorporated into genome-scale flux analysis [33,34], and cellular-economics models showed how growth-rate optimisation can generate trade-offs between metabolic rate and efficiency [35]. Biothermodynamic analyses of microbial growth likewise relate energetic dissipation to growth yield and metabolic driving force [36,37].

Related work has connected population change, information, and thermodynamics through Price-equation formulations [38,39,40], dissipative-adaptation frameworks [41,42,43], thermodynamic bounds on Darwinian selection in molecular replicators [44], and information-theoretic bounds on replicator productivity in flow reactors [45]. At the biomolecular and enzyme scales, recent work has emphasised how performance, evolutionary constraints, and dissipation can be coupled [46,47].

A central opportunity in translating thermodynamic ideas to biological evolution is to represent the actual mechanisms of biological change. In evolving populations, state changes proceed through mutation, selection, and ecological feedback under physiological and biochemical constraints. The present work addresses this issue in an open chemostat, a canonical model of microbial resource competition [48] and an important operating mode in continuous bioprocessing. Controlled long-term microbial cultures are also central to experimental evolution, although the serial-transfer setting of the Long-Term Evolution Experiment is distinct from a chemostat [49].

Our model explicitly incorporates (i) Michaelis–Menten uptake kinetics, grounded in a thermodynamically consistent mesoscopic Chemical Reaction Network (CRN), and (ii) a continuum mutation–selection description in which the replicator equation is supplemented by mutational diffusion, yielding a Crow–Kimura equation. Accordingly, we refer to this framework as mutation–selection dynamics.

The primary aim of our study is to determine the mathematical and biological conditions under which an evolving chemostat population monotonically increases the *metabolic component* of entropy production, and to identify how biochemical constraints or loss of ecological–evolutionary timescale separation alter that tendency. This question sits within the broader debate on the MEPP and biological evolution [50,51]. Our contribution to that debate is a trajectory law for a specified open-system model, with its scope fixed by explicit ecological and thermodynamic assumptions.

We show that monotonic increase follows on the quasi-static ecological manifold and is driven by uptake-rate variance. An exact variance identity, together with a closed solution of the stated low-nutrient, quasi-static, zero-skewness reduction, yields the ideal mass-balance ceiling ΔSJ. Biochemical trade-offs instead generate finite, model-dependent sub-ceilings. When ecological and evolutionary timescales become comparable, a frozen-time Routh–Hurwitz analysis identifies an instantaneous spectral instability boundary. Because the evolutionary variables continue to drift, this boundary is interpreted as a local spectral diagnostic.

The paper is organised as follows. Section 2 defines the dynamical system, the timescale separation parameter, and the entropy balance of the open chemostat. Section 3 grounds the macroscopic Michaelis–Menten kinetics in a mesoscopic, thermodynamically consistent CRN. Section 4, Section 5 and Section 6 develop the ecological equilibrium, establish Lemma 1, and prove Theorem 1. Section 7 derives the continuum mutation–selection model and its exact variance identity, and Section 8 solves the stated closed reduced moment system (Theorems 2 and 3). Section 9 analyses the fast-evolution regime through an instantaneous Routh–Hurwitz spectral criterion and assesses its scope and self-consistency. Section 10 places the results in the Price/Fisher, molecular-replicator, enzyme/metabolic-thermodynamics, and eco-evolutionary literature. Appendix A contains the complete notation table; Appendix B derives the CRN thermodynamics of Section 3 in full; Appendix C and Appendix D contain the remaining detailed algebraic derivations.

## 2. Model Definition

### 2.1. The Chemostat System

We consider a well-mixed continuous-flow bioreactor (chemostat) of fixed volume. This represents a controlled ecosystem where a nutrient is supplied at constant volumetric influx rate *J* (g L^−1^ s^−1^) and physically removed at rate κ (s^−1^). The chemostat contains *n* microbial strains competing for this nutrient. Strain *i* has biomass density $x_{i}\left(t\right)$ (g L^−1^), and the nutrient concentration in the reactor is C(t) (g L^−1^).

To capture the physical reality of transporter saturation, nutrient uptake by strain *i* follows a specific (per-unit-biomass) Michaelis–Menten rate law:

(1) $$\varphi_{i}\left(C\right)=\frac{v_{\max,i}\;C}{K_{m,i}+C},$$

where $v_{\max,i}$ (s^−1^) is the maximum specific uptake rate and $K_{m,i}$ (g L^−1^) is the half-saturation constant. We emphasise that $v_{\max,i}$ here is a specific (mass-normalised) rate, dimensionally analogous to a catalytic turnover number $k_{\mathrm{cat}}$. The macroscopic $V_{\max}$ of classical enzyme kinetics instead includes enzyme abundance. Section 3 makes this normalisation explicit. Each unit of nutrient consumed produces γ (g g^−1^) units of biomass and contributes a specific metabolic entropy yield ΔS (J K^−1^ g^−1^), which we take to be approximately the same for all phenotypes—a closure justified from the underlying reaction-network thermodynamics in Section “Connection Between CRN Entropy Production and the Metabolic Entropy-Production Rate”.

The complete notation of all quantities analysed and discussed in the paper is collected in Table A1 in Appendix A.

### 2.2. Mass-Balance Equations

The coupled ecological dynamics are governed by

(2) $$\frac{dC}{dt}=J-\kappa C-\sum_{i=1}^{n}\varphi_{i}\left(C\right)\;x_{i},$$

(3) $$\frac{dx_{i}}{dt}=\left[\gamma \;\varphi_{i}\left(C\right)-d\right]\;x_{i},\;i=1,\ldots ,n,$$

where $d=d_{w}+d_{m}$ (s^−1^) is the total biomass-removal rate, split into physical washout $d_{w}$ and an internal mortality/turnover rate $d_{m}$. For the standard single-outflow, perfectly mixed chemostat considered here, the same outflow that removes nutrient also removes biomass; therefore, $d_{w}=\kappa$ and, hence, $d=\kappa +d_{m}$. Only $d_{w}=\kappa$ contributes to the boundary entropy flux, while $d_{m}$ is an internal process. With total biomass $X\left(t\right)=\sum_{i}x_{i}$ and relative frequency $p_{i}\left(t\right)=x_{i}/X$, the total population dynamics are

(4) $$\frac{dX}{dt}=\left[\gamma \;\bar{\varphi }\left(C,t\right)-d\right]\;X,\;\bar{\varphi }\left(C,t\right)=\sum_{i}p_{i}\left(t\right)\;\varphi_{i}\left(C\right).$$

These equations describe the supply–consumption–washout balance of a continuous culture. The nutrient concentration is an ecological variable that responds to the aggregate demand of the population. A phenotype that takes up nutrients rapidly can therefore increase its own growth only by changing the environment experienced by all strains. This resource-mediated feedback is the ecological mechanism that later links selection to changes in entropy production.

### 2.3. Entropy Balance and the Metabolic Entropy Production Rate

Let $S_{\mathrm{sys}}$ denote the entropy contained in the reactor per unit reactor volume. With heat input ${\dot{Q}}_{\mathrm{in}}>0$ defined as heat transferred from the environment to the reactor, the entropy balance [52] is

(5) $$\frac{dS_{\mathrm{sys}}}{dt}=\sigma_{\mathrm{int}}+{\dot{S}}_{\mathrm{in}}-{\dot{S}}_{\mathrm{out}}+\frac{{\dot{Q}}_{\mathrm{in}}}{T_{\mathrm{env}}},\;\sigma_{\mathrm{int}}\geq 0,$$

where $\sigma_{\mathrm{int}}$ is the total internal entropy-production rate per unit reactor volume. The material entropy inflow and outflow are

(6) $${\dot{S}}_{\mathrm{in}}=J\;s_{\mathrm{feed}},$$

(7) $${\dot{S}}_{\mathrm{out}}=\kappa \;C\;s_{C}+d_{w}\;X\;s_{X}+{\dot{S}}_{\mathrm{other},\mathrm{out}}=\kappa \;C\;s_{C}+\kappa \;X\;s_{X}+{\dot{S}}_{\mathrm{other},\mathrm{out}},$$

where ${\dot{S}}_{\mathrm{other},\mathrm{out}}$ represents any additional matter streams not retained in the reduced ecological model. In the standard single-outflow construction, $d_{w}=\kappa$ and $d_{m}=d-\kappa$. The first term represents physical export, while the mortality/turnover contribution is internal to the reduced system.

The total internal entropy production may be decomposed schematically as

(8) $$\sigma_{\mathrm{int}}=\sigma_{\mathrm{met}}+\sigma_{\mathrm{mix}}+\sigma_{\mathrm{diff}}+\sigma_{\mathrm{other}},$$

where $\sigma_{\mathrm{mix}}$ and $\sigma_{\mathrm{diff}}$ are background contributions (mixing, diffusion, mortality, and intracellular side reactions) that are treated as background contributions outside the reduced model. The state-dependent quantity analysed here is the metabolic component $\sigma_{\mathrm{met}}$.

For the reduced model, we assign a specific entropy yield ΔS>0 (J K^−1^ g^−1^) to each gram of nutrient metabolised, and we assume that this yield is approximately independent of the phenotype—a closure that becomes a controlled approximation when all strains share a common catabolic pathway and the concentration-dependent part of the thermodynamic affinity is small compared with the standard-state free-energy drop, being exact only in the limiting case in which $A_{i}/T$ is state-independent (the CRN derivation and the precise conditions are given in Section “Connection Between CRN Entropy Production and the Metabolic Entropy-Production Rate”). Under this closure,

(9) $$\sigma \left(t\right)\equiv \sigma_{\mathrm{met}}\left(t\right)=\Delta S\sum_{i}\varphi_{i}\left(C\right)\;x_{i}=\Delta S\;\bar{\varphi }\left(C,t\right)\;X\left(t\right).$$

The dimensions are $\left[\Delta S\right]\left[\bar{\varphi }\right]\left[X\right]=J\;K^{-1}\;L^{-1}\;s^{-1}$. Equation (9) defines the metabolic component of the reactor’s internal entropy production—the product of reaction affinity and reaction flux, in the sense of the chemical-reaction-network thermodynamics literature [10,11,23,24]—and serves as the paper’s evolutionary observable. The assumption that ΔS is phenotype-independent is a deliberate idealisation. Allowing it to vary across strains would couple σ(t) to the full phenotype distribution. A first-principles treatment of the boundary terms is a natural extension that we leave to future work.

### 2.4. Timescale Separation and the Eco-Evolutionary Regimes

The full eco-evolutionary model operates on two distinct fundamental timescales: the ecological relaxation time $\tau_{\mathrm{eco}}∼1/d$ and the evolutionary drift time $\tau_{\mathrm{evo}}∼\bar{\varphi }/\left(\gamma \;\mathrm{Var}\;\varphi \right)$. The dimensionless timescale separation parameter is

(10) $$\delta \equiv \frac{\tau_{\mathrm{eco}}}{\tau_{\mathrm{evo}}}=\frac{\gamma \;\mathrm{Var}\;\varphi }{d\;\bar{\varphi }}.$$

Because δ is built from the population’s own instantaneous variance and mean uptake rate, rather than fixed by an external control, it provides a state-dependent diagnostic of the current timescale separation. The value of δ partitions the dynamical landscape into three regimes:

1. **Quasi-Static Evolutionary Regime** (δ≪1). Ecology equilibrates much faster than evolution. Under the usual smoothness and normal-hyperbolicity assumptions of singular-perturbation theory, the ecological variables relax rapidly toward a slow manifold $M_{0}$. On this manifold, the selection-driven increase in the mean uptake rate is governed by an exact Fisher-type identity (Lemma 1), and the equilibrium entropy-production rate increases monotonically (Theorem 1). When the additional continuum and moment-closure assumptions (Assumption 1, see below) are imposed, the moment equations admit an exact closed solution (Theorems 2 and 3).
2. **Coupled Eco-Evolutionary Regime** (δ∼1). Ecological and evolutionary change occur on comparable timescales, a situation broadly documented in rapid-evolution studies [53,54]. Microbial phenotype-distribution models provide a complementary setting in which substantial phenotypic variability can alter population responses [55]. The full coupled system must then be retained. The instantaneous spectral criterion of Section 9 provides a local frozen-time diagnostic for this regime.
3. **Fast-Evolution Regime** (δ≫1). The evolutionary composition changes on a timescale shorter than ecological relaxation. In the present unbounded linear-trait model, the mean and, under the zero-skewness closure, the variance continue to drift. The relevant description is therefore the full nonstationary coupled dynamics, with the phenotype distribution treated as an evolving state variable.

These regimes have a direct biological interpretation. In the quasi-static regime, a microbial population experiences the environment as nearly equilibrated while it evolves. Near δ∼1, changes in uptake phenotypes can occur on the same timescale as nutrient depletion and biomass turnover; thus, ecology and evolution must be followed together. For δ≫1, the phenotype distribution changes faster than ecological relaxation, and the environment responds with an effective delay. The analysis therefore follows the coupled dynamics directly.

## 3. Thermodynamic Grounding of Michaelis–Menten Kinetics via Chemical Reaction Networks

We now show that Equation (1) is the leading-order forward-flux limit of a thermodynamically consistent CRN, following the approach of Seifert [10] and Rao and Esposito [11]. The reaction scheme below should be understood as a mesoscopic, coarse-grained thermodynamic representation of the uptake process. Its states represent effective biological coordinates at the mesoscopic level. Rather than track every microscopic intracellular step, we treat the cellular growth cycle as a transition between effective states in a mesoscopic state space (e.g., from a nutrient-bound state to a biomass-incremented state). The “species” *C*, $E_{i}$, $EC_{i}$, and $x_{i}$ act as coarse-grained collective variables or internal coordinates that describe the aggregate state of a representative cell or of the population as a whole and are defined independently of individual molecular counts. This interpretation is consistent with the framework of Mesoscopic Non-Equilibrium Thermodynamics (MNET) developed by Reguera, Rubí and Vilar [56], which establishes how entropy production and thermodynamic forces can be consistently formulated over mesoscopic coordinates that do not correspond to conventional, literal molecular species.

Strain *i* takes up the nutrient through a two-step reversible cycle with a surface enzyme/receptor $E_{i}$, $C+E_{i}⇌EC_{i}⇌\mathrm{biomass}+E_{i}$, subject to Local Detailed Balance. Appendix B derives, from the molar chemical potentials of this cycle, the exact thermodynamic affinity $A_{i}^{\mathrm{mol}}$ and the corresponding reversible net uptake flux (Equations (A1)–(A5)). In the biologically relevant forward-flux regime, $A_{i}^{\mathrm{mol}}\gg RT$ and $\left(k_{-2,i}/k_{1,i}\right)\;x_{i}^{\left(a\right)}\ll K_{i}+c$, that flux reduces to

(11) $$\varphi_{i}\left(C,x_{i}\right)≃\frac{v_{\max,i}\;C}{K_{m,i}+C}=\varphi_{i}\left(C\right),\;A_{i}^{\mathrm{mol}}\gg RT,\;\frac{k_{-2,i}\;x_{i}^{\left(a\right)}}{k_{1,i}}\ll K_{i}+c.$$

The MM kinetics used throughout this paper are therefore the leading-order forward-flux limit of a biomass-normalised CRN satisfying Local Detailed Balance.

Note also that the reverse-reaction term (Appendix B) uses the activity-equivalent biomass proxy as the coarse-grained product variable. Of course, accumulated cellular biomass is not itself a small molecule diffusing through the solution, and so this term represents an effective mesoscopic closure at the population level. The reduced ecological model depends on the forward specific uptake function $\varphi_{i}\left(C\right)$. A more detailed representation could replace $x_{i}^{\left(a\right)}$ with an explicit intracellular product pool, a natural extension of the present mesoscopic model.

### Connection Between CRN Entropy Production and the Metabolic Entropy-Production Rate

We now make explicit how the macroscopic metabolic entropy-production rate σ(t) of Equation (9) emerges from the CRN thermodynamics developed above. For a single reaction ρ with molar net flux density $J_{\rho }^{\mathrm{mol}}$ and molar thermodynamic affinity $A_{\rho }^{\mathrm{mol}}$, the local entropy production density is

(12) $$\sigma_{\rho }=\frac{J_{\rho }^{\mathrm{mol}}\;A_{\rho }^{\mathrm{mol}}}{T}.$$

Under the quasi-steady-state reduction for the enzyme-bound intermediate $EC_{i}$, the net flux through the binding step and the net flux through the catalytic step become equal to leading order; thus, a single flux passes through the whole two-step cycle, and the overall uptake cycle can be assigned the cycle affinity $A_{i}^{\mathrm{mol}}$. The exact network expression is the starting point. After the molar affinity is converted to a mass-specific affinity $A_{i}=A_{i}^{\mathrm{mol}}/M_{C}$, the ecological approximation adds the separate closure that the entropy yield per unit nutrient is approximately constant over the operating range:

(13) $$\frac{A_{i}}{T}\approx \Delta S,$$

where ΔS (J K^−1^ g^−1^) is the specific entropy yield per gram of nutrient metabolised. A common standard free-energy change across phenotypes makes the closure more plausible. State dependence can nevertheless remain because chemical potentials vary with the instantaneous concentrations. We therefore treat Equation (13) as an explicit coarse-grained modelling assumption. Under forward-flux dominance and this closure, summing over all strains and using $J_{\mathrm{met},i}^{n}≃\varphi_{i}\left(C\right)\;x_{i}$ gives

(14) $$\sigma_{\mathrm{met}}\left(t\right)=\sum_{i}\frac{J_{\mathrm{met},i}^{n}\;A_{i}}{T}\approx \Delta S\sum_{i}\varphi_{i}\left(C\right)\;x_{i}=\Delta S\;\bar{\varphi }\left(C,t\right)\;X\left(t\right),$$

which recovers Equation (9). Therefore, Equation (9) is the forward-flux, phenotype-independent-ΔS coarse-grained reduction of the exact CRN entropy production in Equation (12). The reduction uses the nonlinear CRN flux directly. Its approximation requires forward-flux dominance together with the separate constant-ΔS closure. We note that this identification would no longer hold if different strains used fundamentally different metabolic pathways (e.g., aerobic versus anaerobic respiration), in which case ΔS would itself become phenotype-dependent. The present model considers a single limiting nutrient processed via a common catabolic route, and so ΔS is naturally constant across strains.

From a biological and ecological perspective, the CRN reduction connects a familiar uptake law to a thermodynamic bookkeeping quantity while retaining the nutrient-processing flux and its driving force. The remaining intracellular details are coarse-grained because the ecological question depends on these effective flux variables rather than on a complete intracellular reaction map.

## 4. Ecological Equilibrium and Local Stability

For a fixed phenotype composition $p=(p_{1},\ldots ,p_{n})$, define $\bar{\varphi }\left(C\right)=\sum_{i}p_{i}\;\varphi_{i}\left(C\right)$. An interior ecological equilibrium satisfies

(15) $$\bar{\varphi }\left(C^{*}\right)=\frac{d}{\gamma },\;X^{*}=\frac{\gamma \;(J-\kappa C^{*})}{d},$$

with $C^{*}>0$ and $J>\kappa C^{*}$. Existence requires that the target rate d/γ lie below the maximum of $\bar{\varphi }\left(C\right)$ over the accessible nutrient range.

For compact closed-form calculations, one may approximate the population-averaged uptake as an effective Michaelis–Menten form $\bar{v}\;C/\left(\bar{K}+C\right)$, where $\bar{v}=\sum_{i}p_{i}\;v_{\max,i}$ and $\bar{K}=\bar{v}/\left[\sum_{i}p_{i}\;\left(v_{\max,i}/K_{m,i}\right)\right]$. Under that approximation,

(16) $$C_{\mathrm{eff}}^{*}=\frac{\bar{K}\;d}{\gamma \;\bar{v}-d}.$$

The exact local derivative, used in the Jacobian below and again in the proof of the monotonicity theorem of Section 6, is

(17) $$\alpha \equiv {\left.\frac{\partial \bar{\varphi }}{\partial C}\right|}_{C^{*}}=\sum_{i}p_{i}\;\frac{v_{\max,i}\;K_{m,i}}{{\left(K_{m,i}+C^{*}\right)}^{2}}>0.$$

The ecological Jacobian is

(18) $$J_{\mathrm{eco}}=\left(\begin{array}{cc} -(\kappa +\alpha X^{*}) & -d/\gamma \\ \gamma \alpha X^{*} & 0 \end{array}\right),\;\mathrm{Tr}\;J_{\mathrm{eco}}<0,\;\det\;J_{\mathrm{eco}}=\alpha \;d\;X^{*}>0.$$

Hence, any positive interior ecological equilibrium is locally asymptotically stable for fixed composition.

This stability can be interpreted as the restoring feedback expected in a nutrient-limited culture: if the nutrient concentration rises, uptake and growth increase and biomass draws the nutrient back down; if the nutrient concentration falls, growth slows and washout reduces demand. The ecological subsystem therefore acts as a stabilising background against which evolutionary changes in phenotype can be distinguished from ordinary resource fluctuations.

## 5. Replicator Dynamics Under Michaelis–Menten Kinetics

We now allow the phenotype frequencies $p_{i}$ to evolve under selection, while the nutrient concentration *C* is still governed by the ecological dynamics illustrated in Section 4. The discrete strain model is retained, and the key new variable is the fitness variance Varφ(C,t).

### 5.1. Derivation of the Replicator Equation

The dynamics of the phenotype frequencies follow directly from the strain-level mass-balance equations above. This frequency transformation introduces no additional biological assumption. Using the quotient rule on $p_{i}=x_{i}/X$,

(19) $${\dot{p}}_{i}=\gamma \;p_{i}\;\left[\varphi_{i}\left(C\right)-\bar{\varphi }\left(C\right)\right],$$

structurally identical to the standard replicator equation of evolutionary game theory [57].

### 5.2. Statement and Proof of Lemma 1

**Lemma 1** 
(Fisher-type decomposition of the mean uptake rate)**.** *Let $\bar{\varphi }\left(C,t\right)=\sum_{i}p_{i}\left(t\right)\;\varphi_{i}\left(C\left(t\right)\right)$. The selection-driven component of its rate of change, holding the environment C fixed, is exactly*

(20) $$\sum_{i}{\dot{p}}_{i}\;\varphi_{i}\left(C\right)=\gamma \;\mathrm{Var}\;\varphi \left(C,t\right),$$

*while the total time derivative, including ecological feedback, is*

(21) $$\frac{d\bar{\varphi }}{dt}=\gamma \;\mathrm{Var}\;\varphi \left(C,t\right)+\sum_{i}p_{i}\;\frac{\partial \varphi_{i}}{\partial C}\left(C\right)\;\dot{C}.$$

*Both relations are exact for any trajectory C(t). On the ecological equilibrium manifold $M_{0}$, where $\bar{\varphi }\left(C^{*}\left(p\right),p\right)=d/\gamma$ identically, the left-hand side of *(21) *vanishes, forcing the ecological-feedback term to exactly cancel the selection term γVarφ.*

**Proof.**  See Appendix C. □

Equation (20) shows that standing differences in nutrient uptake are the immediate fuel for selection. If some strains acquire the limiting nutrient faster than others, their relative abundance increases. When all co-existing strains have nearly identical uptake rates at the current nutrient concentration, the uptake-based selection differential becomes small and adaptation slows. At the same time, successful strains reduce the nutrient concentration, and so the environment feeds back on the absolute performance of every phenotype.

The covariance identity is an established evolutionary result whose role here is structural. It isolates the exact cancellation between selection and ecological feedback on the equilibrium manifold, which is the mechanism underlying Theorem 1. Its thermodynamic use differs from earlier Price/Fisher treatments because the entropy production observable is independently defined and thermodynamically grounded in Section 3 [38,39,40,58].

## 6. Entropy Production at Ecological Equilibrium

### 6.1. Expression for $\sigma^{*}$ and Nonlinear Averaging Approximation

At ecological equilibrium, the entropy production rate is

(22) $$\sigma^{*}=\Delta S\cdot \bar{\varphi }\left(C^{*}\right)\cdot X^{*}=\Delta S\cdot \left(J-\kappa C^{*}\right).$$

This is the metabolic contribution to the internal entropy production $\sigma_{\mathrm{int}}$. The exchange terms in Equation (5) are accounted for separately through the boundary conditions. Because a weighted average of heterogeneous Michaelis–Menten curves is not, in general, itself an exact Michaelis–Menten curve, we use an effective two-parameter representation only for the following compact estimate. The chosen $\bar{v}$ and $\bar{K}$ match the population’s high-concentration plateau and low-concentration slope, respectively. Equation (23) is therefore used only as an approximation and does not enter the exact monotonicity result.

(23) $$\sigma^{*}\left(\bar{v},\bar{K}\right)\approx \Delta S\;\left(J-\kappa \;\frac{\bar{K}\;d}{\gamma \;\bar{v}-d}\right).$$

### 6.2. Temporal Monotonicity of $\sigma^{*}$

**Theorem 1** 
(Temporal monotonicity of equilibrium entropy production)**.** *Along the evolutionary trajectory on the slow manifold $M_{0}$, the equilibrium entropy production rate satisfies*

(24) $$\frac{d\sigma^{*}}{dt}=\frac{\Delta S\;\kappa \;\gamma }{\alpha }\;\mathrm{Var}\;\varphi \left(C^{*},t\right)\geq 0,$$

*where α is the exact derivative defined in Equation *(17)*. The inequality is strict (${\dot{\sigma }}^{*}>0$) whenever the population has positive uptake-rate variance (Varφ>0). Polymorphism alone does not guarantee a strict increase if distinct phenotypes have the same uptake rate at the current nutrient concentration.*

**Proof.**  See Appendix C. □

The monotonicity theorem illustrates how selection shifts biomass toward strains that acquire the limiting nutrient more effectively. On the ecological equilibrium manifold, this lowers the nutrient concentration required to sustain the same biomass-loss rate. Less of the incoming nutrient therefore leaves through nutrient washout and more is processed by the population, causing the metabolic entropy-production rate to rise. The theorem therefore shows how ordinary resource competition generates a measurable thermodynamic consequence at the population level.

**Monotonicity versus extremum principle.** Theorem 1 establishes a monotonic increase in $\sigma^{*}$ along the evolutionary trajectory. The result is therefore dynamical rather than variational: it constrains how $\sigma^{*}$ changes in time but does not identify the endpoint as the solution of an optimisation problem. A variational principle would make the stronger claim that the attained state solves an independently defined optimisation problem [2]. Entropy production has also been shown to enter structure-selection criteria in non-equilibrium self-assembly without invoking a universal extremum principle [59]. In the same spirit, our result identifies a selection-driven thermodynamic tendency while leaving global variational characterisation as a separate question.

### 6.3. Effect of Phenotypic Trade-Offs

Consider the phenomenological power-law trade-off $v_{\max}=A_{\mathrm{tr}}\;K_{m}^{\beta }$ with 0<β<1. Introducing $k=v_{\max}/K_{m}$ and q=1/(1−β)>1 gives

(25) $$K_{m}\left(k\right)={\left(\frac{A_{\mathrm{tr}}}{k}\right)}^{q},\;v_{\max}\left(k\right)=A_{\mathrm{tr}}^{q}\;k^{-\beta q},$$

and therefore the full Michaelis–Menten uptake rate becomes

(26) $$U\left(k,C\right)\equiv \varphi \left(k,C\right)=\frac{C\;k}{1+\left(C/A_{\mathrm{tr}}^{q}\right)\;k^{q}}.$$

For every fixed C>0, U(k,C)→0 as $k\to 0^{+}$ and as k→∞; therefore, *U* has a unique interior maximum because its derivative changes sign once. Its location is

(27) $$k^{†}\left(C\right)={\left[\frac{A_{\mathrm{tr}}^{q}}{C\;(q-1)}\right]}^{1/q}.$$

The optimum is therefore environment-dependent. In an eco-evolutionary equilibrium, $C^{†}$ and $k^{†}$ must be determined jointly from $U^{\prime }\left(k^{†},C^{†}\right)=0$, $\gamma \;U(k^{†},C^{†})=d$, and the nutrient balance.

A local quadratic expansion around the interior optimum, $U\left(k,C^{*}\right)\approx U^{*}-M_{U}\;{\left(k-k^{†}\right)}^{2}/2$ with $M_{U}=-\partial_{k}^{2}U\left(k^{†},C^{*}\right)>0$, provides a standard stabilising-selection picture. Here, $M_{U}$ is the curvature of the full specific uptake rate U=φ at fixed $C^{*}$ and therefore already contains the nutrient concentration dependence. Under an additional approximately Gaussian, mean-centred closure for the phenotype distribution, this curvature produces a variance equation of the form

(28) $$\frac{d\;\mathrm{Var}}{dt}\approx -\gamma \;M_{U}\;{\mathrm{Var}}^{2}+2D_{\mu },$$

with local stationary variance

(29) $${\mathrm{Var}}^{†}\approx \sqrt{\frac{2D_{\mu }}{\gamma \;M_{U}}}.$$

Equation (28) is a local moment closure derived from a quadratic fitness expansion and an approximately Gaussian, mean-centred phenotype distribution. It is the biochemical analogue of Lande–Turelli mutation–selection balance [60,61], stated here under a local quadratic-fitness-plus-fourth-moment closure. It complements, rather than continues, the unbounded linear-trait model of Section 7 and Section 8, representing instead the stabilising biochemical curvature of a separate bounded scenario.

The monotonicity theorem continues to apply to the discrete trade-off model because it depends on uptake-rate variance, not on the absence of constraints. In the monomorphic or narrow-distribution limit, stabilising selection drives the mean toward the interior optimum and gives the leading-order sub-ceiling $\sigma_{\infty }^{*}\left(\beta \right)=\Delta S\left(J-\kappa C^{†}\right)<\Delta SJ$, with $C^{†}=dq/\left[\gamma k^{†}\left(q-1\right)\right]$. For a population with finite stationary variance, $\bar{\varphi }$ is not exactly $U(\bar{k},C)$, and so the equilibrium nutrient concentration and sub-ceiling acquire moment corrections. The free-trait continuum model of Section 7 and Section 8 is a separate modelling scenario rather than a parameter limit of this trade-off model.

From an ecological perspective, the trade-off calculation captures a familiar point. Increasing uptake performance is rarely free. A faster transporter, enzyme, or receptor system can carry costs in protein allocation, specificity, folding, regulation, or energy use. The mathematical consequence is that the phenotype with the largest instantaneous nutrient uptake can lie at an interior point of trait space. The population can therefore approach a compromise in which marginal gains from further kinetic investment disappear. This is why the trade-off model produces a finite sub-ceiling rather than the unbounded limit of the free-trait model. The local stability implications of this interior optimum for the fast-evolution regime are analysed in Section 9.2.

## 7. The Continuum Limit of Selection: A Reaction–Diffusion Equation

We now move from the discrete strain model of Section 2, Section 3, Section 4, Section 5 and Section 6 to a continuous phenotype density ρ(k,t) over the trait $k=v_{\max}/K_{m}$. Lemma 1 and Theorem 1 describe adaptation within a population of co-existing but fixed strains: selection reshapes their relative frequencies within the existing phenotype set. Without mutation, Varφ→0, and both adaptation and the entropy-production increase of Theorem 1 cease, as the variance that fuels adaptation is exhausted. Mutation supplies the additional source of phenotypic exploration needed to sustain long-term evolutionary dynamics, and so we embed the selection process in a continuum phenotype model. The key assumptions of this and the following two sections are stated in Assumption 1 below. Note that Var(k,t) here denotes continuous phenotypic variance, distinct from the discrete uptake-rate variance Varφ used above.

**Assumption 1** 
(Linear-kinetics regime)**.** *Throughout Section 7, Section 8 and Section 9, we work in the low-nutrient limit $C\ll K_{m}$, where φ(k,C)≈kC. For analytical convenience, the positive trait $k=v_{\max}/K_{m}$ is extended to k∈R. The intended biological distributions are concentrated on k>0. A strictly biological formulation would instead use k≥0 with an appropriate boundary condition at k=0. The moment closure $\mu_{3}\approx 0$ is imposed only when the closed solution is derived. Under these assumptions, the moment equations derived below (Theorems 2 and 3) admit an exact closed solution under the stated closure. A full Michaelis–Menten treatment would instead retain the nonlinear fitness function throughout the population dynamics. This generally couples the moment hierarchy to higher-order moments and requires additional closure information beyond the level used here, providing a natural direction for future work.*

### 7.1. Continuous Phenotype Distribution

Let ρ(k,t) be a density over k∈(−∞,∞) with ∫ρdk=1, $\bar{k}=\int k\;\rho \;dk$, $\mathrm{Var}\left(k,t\right)=\int {\left(k-\bar{k}\right)}^{2}\;\rho \;dk$. We assume that ρ and its derivatives decay sufficiently rapidly as |k|→∞ that the integration-by-parts boundary terms used below vanish.

### 7.2. Derivation from the Discrete Replicator Equation

The correct continuum limit of the selection term, in the linear-kinetics regime φ(k,C)=kC, is the pure reaction (multiplicative growth) term:

(30) $${\left.\frac{\partial \rho }{\partial t}\right|}_{\mathrm{sel}}=\gamma \;C\;\left(k-\bar{k}\right)\;\rho .$$

Adding mutation as diffusion in phenotype space yields the continuous-time Crow–Kimura mutation–selection equation [55,62]:

(31) $$\frac{\partial \rho }{\partial t}=\gamma \;C\;\left(k-\bar{k}\right)\;\rho +D_{\mu }\;\frac{\partial^{2}\rho }{\partial k^{2}}.$$

The Crow–Kimura equation separates two biological roles. Selection changes the relative abundance of existing phenotypes, whereas mutation moves the population through phenotype space and continually supplies new variation. The low-nutrient approximation specifies the nutrient-limited operating regime in which the analytical reduction applies.

### 7.3. Exact Variance Equation

**Theorem 2** 
(Exact variance equation)**.** *Within the Crow–Kimura model and the stated boundary conditions, the phenotypic variance exactly obeys*

(32) $$\frac{d\;\mathrm{Var}(k,t)}{dt}=\gamma \;C\left(t\right)\;\mu_{3}\left(t\right)+2D_{\mu },$$

*where $\mu_{3}\left(t\right)=\int {\left(k-\bar{k}\right)}^{3}\;\rho \;dk$. Under the zero-skewness closure $\mu_{3}\approx 0$, the variance follows $d\;\mathrm{Var}/dt=2D_{\mu }>0$ and therefore grows linearly in time. For arbitrary non-Gaussian initial data, $\mu_{3}=0$ is a closure rather than a dynamically invariant property, and the exact equation retains the skewness term. For Gaussian initial data, however, the linear-fitness, constant-diffusion Crow–Kimura equation preserves Gaussianity, and so zero skewness and $d\;\mathrm{Var}/dt=2D_{\mu }$ are exact within that Gaussian family.*

**Proof.**  See Appendix C. □

The unbounded variance equation provides a useful analytical reference case. Mutation continually injects phenotypic spread, while stabilising selection, finite trait ranges, energetic costs, and other biological constraints can counteract this tendency. Retaining the exact skewness term shows explicitly how departures from the closed linear-growth result arise in broader phenotype distributions.

**Corollary 1** 
(Stabilising selection under phenotypic trade-offs)**.** *When the specific growth function acquires a localised negative curvature $M_{u}>0$ at the phenotypic optimum, a quadratic expansion $f\left(k\right)=f^{*}-\frac{M_{u}}{2}{\left(k-k^{*}\right)}^{2}+\cdots$ gives variance dynamics that, under the quasi-Gaussian moment closure $\mu_{4}\approx 3\;{\mathrm{Var}}^{2}$, acquire a saturating nonlinearity:*

(33) $$\frac{d\;\mathrm{Var}}{dt}=-\gamma \;C\left(t\right)\;M_{u}\;{\mathrm{Var}}^{2}+2D_{\mu },$$

*with stable fixed point ${\mathrm{Var}}^{†}=\sqrt{2D_{\mu }/\left(\gamma \;C^{*}\;M_{u}\right)}$. This is the biochemical analogue of Lande–Turelli mutation–selection balance [60,61], stated here under a local quadratic-fitness-plus-fourth-moment closure.*

Our analysis implies that the correct continuum limit of selection is a reaction term rather than an advection term. This reflects the fact that selection changes the *frequencies* of existing phenotypes, not the trait values themselves; therefore, only mutation moves the distribution in trait space. The thermodynamic consequence is that selection reshapes the population while preserving the spread of a delocalising distribution (Theorem 2): any bound on phenotypic variance must therefore come from biochemistry, rather than from selection dynamics alone—concretely, the stabilising curvature introduced in the trade-off model of Section 6.3. There, the curvature $M_{U}$ of the full uptake function is evaluated already at the equilibrium nutrient level $C^{*}$, whereas Equation (33) above expresses the same curvature as a bare, per-nutrient quantity $M_{u}$ with the nutrient dependence left explicit as a separate factor C(t); the two are related by $M_{U}=C^{*}\;M_{u}$, and so the stationary variances coincide: ${\mathrm{Var}}^{†}=\sqrt{2D_{\mu }/\left(\gamma M_{U}\right)}$ (Equation (29)) $=\sqrt{2D_{\mu }/\left(\gamma \;C^{*}\;M_{u}\right)}$, replacing the unbounded linear growth of the variance in the free-trait model.

## 8. Exact Solution of the Moment System and Asymptotic Entropy Production

Remaining in the continuous phenotype description of Section 7 and the linear-kinetics regime of Assumption 1, the quasi-static ecological approximation $C\left(t\right)\approx C^{*}$ and the moment closure $\mu_{3}\approx 0$ are now imposed, reducing the Crow–Kimura PDE to a closed pair of ODEs for $\bar{k}\left(t\right)$ and Var(t).

Under the quasi-static approximation $C\left(t\right)\approx C^{*}=d/\left(\gamma \bar{k}\right)$ and $\mu_{3}\approx 0$,

(34) $$\frac{d\bar{k}}{dt}=\frac{d\cdot \mathrm{Var}(t)}{\bar{k}},\;\frac{d\;\mathrm{Var}}{dt}=2D_{\mu }.$$

**Theorem 3** 
(Exact solution and asymptotic entropy production)**.** *For the closed system (34), the equations integrate exactly to give*

(35) $$\bar{k}\left(t\right)=\sqrt{{\bar{k}}_{0}^{2}+2d\;{\mathrm{Var}}_{0}\;t+2d\;D_{\mu }\;t^{2}},$$

*and consequently, $C^{*}\left(t\right)\to 0$ and $\sigma^{*}\left(t\right)\to \Delta S\cdot J$ as t→∞.*

The ceiling ΔSJ is the idealised limit obtained when essentially all of the incoming nutrient is processed by the population and nutrient washout becomes negligible. The unbounded model therefore provides the limiting trajectory against which biochemical or physiological constraints on increasing uptake performance can be measured. It is best interpreted as an analytical benchmark against which finite trade-off ceilings can be compared.

**Proof.**  See Appendix C. □

The solution separates two sources of adaptation within the reduced model: the term proportional to ${\mathrm{Var}}_{0}$ records pre-existing phenotypic variance, whereas the $D_{\mu }t^{2}$ term records variance continually supplied by mutation. The resulting $\sqrt{t}$-to-linear-*t* crossover is therefore a property of this closure and should not be identified with the history of a particular evolution experiment.

## 9. The Fast-Evolution Regime: Eco-Evolutionary Coupling

We now leave the Quasi-Static Evolutionary Regime (δ≪1) and consider the Coupled Eco-Evolutionary Regime (δ∼1), in which ecological and evolutionary timescales are comparable. Section 9.1 then uses a frozen-time linearisation as a diagnostic of the Fast-Evolution Regime (δ≫1). The low-nutrient approximation φ(k,C)=kC is retained.

The coupled model is

(36) $$\dot{C}=J-\kappa C-\bar{k}\;C\;X,\;\dot{X}=\left(\gamma \;\bar{k}\;C-d\right)\;X,$$

and, under the zero-skewness continuum closure,

(37) $$\dot{\bar{k}}=\gamma \;C\;\mathrm{Var},\;\dot{\mathrm{Var}}=2D_{\mu }.$$

Because $\bar{k}$ and Var drift whenever C>0 and $D_{\mu }>0$, the full four-dimensional system is generally nonstationary.

δ∼1 marks the point at which environmental feedback and evolutionary change occur on comparable timescales. A change in the phenotype distribution immediately alters nutrient demand, while the resulting nutrient depletion simultaneously changes the fitness differences that drive selection. The population and its environment can therefore chase one another rather than settle onto a single slow ecological manifold.

### 9.1. Instantaneous Frozen-Parameter Spectral Criterion

For fixed instantaneous values of $\bar{k}>0$ and V=Var>0, consider the instantaneous ecological equilibrium in the (C,X) subsystem defined by $C^{*}=d/\left(\gamma \bar{k}\right)$ and $X^{*}=\left(J-\kappa C^{*}\right)/\left(\bar{k}\;C^{*}\right)$, assuming $X^{*}>0$. Because $\dot{\bar{k}}=\gamma CV$ is nonzero for V>0, this point is not an equilibrium of the full evolutionary system, but instead an instantaneous base state of the evolving system. The matrix $J_{\mathrm{fr}}$ is the Jacobian of the frozen-time variational dynamics evaluated at that drifting state:

(38) $$J_{\mathrm{fr}}=\left(\begin{array}{ccc} -(\kappa +\bar{k}\;X^{*}) & -\bar{k}\;C^{*} & -C^{*}X^{*}\\ \gamma \;\bar{k}\;X^{*} & 0 & \gamma \;C^{*}X^{*}\\ \gamma \;V & 0 & 0 \end{array}\right).$$

Expanding $\det(J_{\mathrm{fr}}-\lambda I)=0$ gives the characteristic polynomial $\lambda^{3}+a_{2}\;\lambda^{2}+a_{1}\;\lambda +a_{0}=0$ with coefficients $a_{2}=\kappa +\bar{k}\;X^{*}$, $a_{1}=\gamma \;C^{*}\;X^{*}\;\left({\bar{k}}^{2}+V\right)$, and $a_{0}=\gamma^{2}\;\bar{k}\;{\left(C^{*}\right)}^{2}\;X^{*}\;V$; the determinant expansion and the simplification of the Routh–Hurwitz margin $a_{2}a_{1}-a_{0}$ using $\gamma \bar{k}C^{*}=d$ are given in Appendix D.

**Proposition 1** 
(Frozen-parameter Routh–Hurwitz instability threshold)**.** *For $a_{2}>0$ and $a_{0}>0$, the frozen cubic is linearly stable when $a_{2}\;a_{1}>a_{0}$. If $\kappa +\bar{k}\;X^{*}<d$, the margin crosses zero at*

(39) $$V_{c}=\frac{\left(\kappa +\bar{k}\;X^{*}\right)\;{\bar{k}}^{2}}{d-\kappa -\bar{k}\;X^{*}}.$$

*At $V=V_{c}$, the frozen cubic has the factorisation $\left(\lambda +a_{2}\right)\left(\lambda^{2}+a_{1}\right)$, and so the linearisation has the pair $\lambda =\pm i\sqrt{a_{1}}$ together with $\lambda =-a_{2}$. For $V>V_{c}$, the frozen linearisation violates the Routh–Hurwitz condition.*

Using $C^{*}=d/\left(\gamma \bar{k}\right)$ and $X^{*}=\gamma J/d-\kappa /\bar{k}$, the existence condition for the threshold can be written exactly as

(40) $$\kappa +\bar{k}X^{*}=\frac{\gamma J\bar{k}}{d}<d\;⟺\;\bar{k}<\frac{d^{2}}{\gamma J}.$$

Positive biomass additionally requires $\bar{k}>\kappa d/\left(\gamma J\right)$. Hence, a non-empty frozen-instability window requires d>κ or, equivalently, a positive internal mortality/turnover rate $d_{m}=d-\kappa >0$. In the ideal chemostat with biomass loss only by washout ($d_{m}=0$), this particular frozen instability cannot occur. Equivalently, the condition is $\bar{k}X^{*}<d_{m}$.

This proposition supplies an instantaneous spectral diagnostic for the coupled feedback. Because $\bar{k}$ and *V* continue to evolve through the threshold, the result locates a frozen-parameter boundary. Establishing a periodic orbit would require a separate autonomous bifurcation analysis.

The criterion is relevant as a mathematical diagnostic for systems in which rapid evolutionary change feeds back to ecology. Such feedback has been demonstrated experimentally in rotifer–algal microcosms [53], and bacteria–phage co-evolution has been observed directly in continuous chemostat culture [63]. These examples provide empirical motivation for the timescale question. The threshold itself is model-specific, and its scope and self-consistency are analysed in Section 9.3.

From a biological perspective, the interpretation of the threshold is deliberately narrow. A broad phenotypic distribution strengthens eco-evolutionary feedback in the frozen calculation, and beyond the threshold, small perturbations grow in the instantaneous linearisation. The subsequent population dynamics depend on how rapidly the mean phenotype and variance continue to change, together with the nonlinear biochemical and ecological constraints of the full system.

### 9.2. Bounded Interior Optima and Boundary-Constrained Traits

The bounded trade-off model of Section 6.3 is distinct from the unbounded linear-trait approximation of Section 7 and Section 8; it is not a formal β→1 limit of that model. To describe an interior optimum in the low-nutrient, locally separable setting, we define the specific uptake, consistent with the low-nutrient linearisation of Assumption 1 applied at each fixed trait value but now allowing the trait-dependent slope u(k) to be an arbitrary function of *k* rather than the linear form u(k)=k used there, as

(41) φ(k,C)≃Cu(k),

where u(k) is the per-nutrient uptake coefficient. The exact Price-equation form is

(42) $$\dot{\bar{k}}=\gamma \;C\;\mathrm{Cov}\left(k,u\left(k\right)\right).$$

A local expansion of *u* about $\bar{k}$ gives the leading-order closure

(43) $$\dot{\bar{k}}\approx \gamma \;C\;u^{\prime }\left(\bar{k}\right)\;\mathrm{Var}\left(k\right);$$

the exact covariance form remains valid without this local approximation. At an interior smooth optimum $k^{†}$, $u^{\prime }\left(k^{†}\right)=0$ and $u^{\prime \prime }\left(k^{†}\right)<0$, and so the leading evolutionary feedback vanishes at first order. For the smooth closure

(44) $$\dot{C}=J-\kappa C-C\;u\left(k\right)\;X,\;\dot{X}=\left(\gamma \;C\;u\left(k\right)-d\right)\;X,\;\dot{k}=\gamma \;C\;\mathrm{Var}\;u^{\prime }\left(k\right),$$

the Jacobian at $k=k^{†}$ is block triangular. Its ecological 2×2 block has negative trace and positive determinant, while the trait eigenvalue is $\gamma \;C^{*}\;{\mathrm{Var}}^{†}\;u^{\prime \prime }\left(k^{†}\right)<0$. Thus, the interior smooth optimum is locally stable, and the first-order oscillatory feedback identified by the frozen linear-trait model is switched off at the optimum.

This result identifies the stabilising mechanism in the bounded model. An interior optimum removes the first-order selection gradient. This distinguishes two different ways a trait can stop changing. In the interior-optimum case just analysed, selection itself weakens and vanishes as the trait approaches its best value—the way ordinary stabilising selection works on a fitness peak—and so the population settles down on its own, with no residual push in either direction. A different situation arises if evolution instead runs up against a hard physical or biochemical limit (for example, a maximum possible enzyme turnover rate) before reaching such a peak: selection would still favour moving further, but the trait simply cannot follow. That is a fundamentally different, boundary-constrained problem, and reaching a hard limit does not by itself guarantee the same stability that an interior fitness peak provides. Such a situation requires a separate constrained-dynamics analysis, which we do not undertake here.

### 9.3. Scope and Self-Consistency of the Frozen-Parameter Instability Criterion

Proposition 1 provides an instantaneous spectral result for the drifting four-dimensional system. Equation (40) makes its self-consistency transparent analytically. For the unbounded linear-trait model, the frozen threshold is available only while

$$\frac{\kappa d}{\gamma J}<\bar{k}<\frac{d^{2}}{\gamma J},$$

where the lower bound is the positive-biomass condition and the upper bound is the Routh–Hurwitz admissibility condition. As continued adaptation increases $\bar{k}$, this admissible interval necessarily closes at the finite value $d^{2}/\left(\gamma J\right)$; at the same time $V_{c}$ diverges as its denominator tends to zero. Determining whether the evolving trajectory reaches $V>V_{c}$ before the window closes therefore requires integration of the nonautonomous coupled dynamics, with $\bar{k}$ and *V* evolving as state variables rather than being treated as independent control parameters.

A different construction is a *mean-capped boundary model*: suppose an external physiological or hard-trait constraint arrests the mean at $k_{\mathrm{cap}}$ while mutational diffusion remains effectively unopposed. If

$$\frac{\kappa d}{\gamma J}<k_{\mathrm{cap}}<\frac{d^{2}}{\gamma J},$$

then $V_{c}$ remains finite, and the idealised law $\dot{V}=2D_{\mu }$ eventually carries the frozen linearisation beyond the Routh–Hurwitz threshold. This construction defines a hard-boundary model and is analysed separately from the smooth interior-optimum trade-off model of Section 6.3.

For a smooth biochemical trade-off, the local curvature represented by Corollary 1 stabilises both the trait mean and the variance. At the interior optimum $u^{\prime }\left(k^{†}\right)=0$, the first-order evolutionary feedback vanishes and the local trait eigenvalue is negative. Whether the finite stationary variance ${\mathrm{Var}}^{†}$ can enter any instability region away from that local optimum requires a fully specified nonlinear model beyond the local information contained in Proposition 1. The analysis therefore distinguishes three cases: an unbounded drifting model, a hard mean-capped model with continuing variance growth, and a smooth stabilising-selection model in which both moments are bounded.

## 10. Discussion and Conclusions

We have developed a tractable open-chemostat model that quantitatively links Darwinian selection, resource competition, and metabolic entropy production. The thermodynamic bookkeeping distinguishes the metabolic contribution from the total entropy balance of the reactor, while the Michaelis–Menten uptake law is grounded in a reversible mesoscopic CRN. Under the explicit forward-flux and approximately phenotype-independent entropy-yield closures, the metabolic observable $\sigma =\Delta S\sum_{i}\varphi_{i}x_{i}$ is therefore connected to independently defined reaction-network thermodynamics.

Within these assumptions, the principal quasi-static result is exact: on the ecological slow manifold, positive variance in specific nutrient uptake produces a monotonic increase in the equilibrium metabolic entropy-production rate. The underlying mechanism is straightforward. Selection changes strain frequencies according to uptake-dependent growth; ecological equilibration then lowers the residual nutrient concentration as resource acquisition improves; and the steady nutrient balance converts this resource drawdown into increased metabolic throughput. These steps yield $\sigma^{*}=\Delta S\left(J-\kappa C^{*}\right)$ and the monotonicity relation in Equation (24). This is a trajectory result for the specified open system, not a statement about the total entropy production of the reactor or a proof that the attained state solves an independently defined variational optimisation problem.

The continuum mutation–selection analysis extends this result by resolving how phenotypic variation is generated and constrained. The variance identity retains the skewness contribution exactly, whereas the closed asymptotic solution additionally requires the low-nutrient, quasi-static, and zero-skewness assumptions; Gaussian initial data provide an exactly preserved family for the latter condition. In the unbounded reference model, the resulting asymptotic value ΔSJ is the mass-balance ceiling. Smooth biochemical trade-offs instead generate finite trait optima and, under the local Gaussian closure, finite mutation–selection variance and a model-dependent sub-ceiling.

A complementary result emerges when ecological and evolutionary timescales become comparable. The frozen-time Routh–Hurwitz calculation identifies an instantaneous spectral-instability boundary and shows that its admissible window requires $d_{m}=d-\kappa >0$. A smooth interior phenotypic optimum suppresses the corresponding first-order evolutionary feedback. The analysis therefore identifies conditions for local linear amplification but does not establish persistent periodic dynamics, whose existence would require analysis of the fully nonlinear eco-evolutionary system.

These results provide a specific mathematical realisation of a long-standing line of thought in evolutionary energetics. Lotka related evolution to energetic throughput [64], and Ulanowicz and Hannon developed an ecosystem-level thermodynamic perspective on life and entropy production [65]. More recent discussions have revisited related ideas in terms of entropy-production tendencies and extremum principles [50,51]. The present chemostat model adds a population-dynamical level to this historical lineage by identifying explicit conditions under which a thermodynamic tendency emerges from mutation, selection, resource competition, and open-system mass balance.

The result also connects naturally with the Price/Fisher literature. Price-equation formulations have related population change to information and entropy descriptions [38,39,40], and Qian derived a related connection between fitness and entropy production in phenotype-switching cell populations [58]. Although the covariance identity itself is standard evolutionary machinery, its significance here lies in the link it provides between levels of description: standing variation in the uptake rate changes the ecological state, and this ecological feedback, in turn, alters a thermodynamic observable independently grounded in CRN thermodynamics. This coupling among selection, resource balance, and metabolic entropy production is the distinctive step leading to Equation (24).

Our analysis is also complementary to thermodynamic treatments of molecular replication. England and collaborators related dissipative driving to self-replication and adaptation [41,42,43]. Kolchinsky derived thermodynamic-affinity bounds on replication fitness and Darwinian selection in autocatalytic molecular replicators, including a steady-state flow-reactor setting [44], and Piñero et al. derived information-theoretic bounds on replicator productivity in fluctuating flow reactors [45]. These approaches quantify how thermodynamic driving or environmental information constrains replication and productivity. The present model addresses a different but complementary question: how resource-mediated selection changes metabolic entropy production along a population-level evolutionary trajectory, and how ecological and biochemical constraints shape its attainable level.

Entropy-production optimisation in enzyme and metabolic systems provides another relevant point of comparison. Dobovišek et al. formulated explicit entropy-production extrema and MEPP-based analyses for enzyme-kinetic models [25,26,27]. Unrean and Srienc combined a reduced metabolic network with adaptive-evolution experiments and reported evolution toward a model-predicted high-entropy-production state [28], and so their result is not simply a fixed-network variational calculation. Recent enzyme-level work has likewise emphasised empirical and theoretical links among catalytic parameters, evolutionary constraints, and dissipation [47].

Taken together, these studies suggest a useful hierarchy of thermodynamic claims. One level concerns monotonic change along an evolutionary trajectory; a second concerns convergence toward an attainable ceiling; and a third asks whether that ceiling solves a separately defined static optimisation problem. The present analysis establishes the first level and, under the reduced unbounded continuum closure, the second. The third belongs to a separate variational programme. This hierarchy allows different entropy-production claims to be compared without conflating dynamical, asymptotic, and variational statements [2,7,8,9].

### 10.1. Biophysical Constraints and Experimental Interpretation

The ideal ceiling ΔSJ is a useful mass-balance benchmark, but physiological constraints will generally place the biologically attainable state below it. Enzyme and transporter phenotypes are shaped by catalytic trade-offs and cellular resource allocation. Accuracy–rate trade-offs are well documented in enzyme evolution [66,67], while bacterial growth laws and protein-expression studies show that increased resource-acquisition capacity competes with allocation to other proteome sectors [68,69]. Quantitative proteomics in steady-state continuous cultures has directly measured growth-dependent proteome reallocation in *E. coli* [70], and broader condition-resolved proteome maps provide complementary evidence [71]. These observations support the use of a stabilising curvature as a coarse-grained representation of biological cost while also identifying fixed γ and fixed receptor abundance as simplifying assumptions that can be relaxed in more mechanistic extensions.

Accordingly, the power-law relation introduced in Section 6.3 should be interpreted as an analytically convenient phenomenological representation of such constraints rather than as a universal enzyme law. At a smooth interior optimum, mutation and stabilising selection can balance at finite variance, and for a finite-width population, the corresponding entropy-production sub-ceiling acquires moment corrections beyond the monomorphic expression. The Pareto structure identified in minimal biomolecular adaptation models [46] and the proposed dissipation landscape for enzyme catalytic parameters [47] provide mechanistic motivation for bounded trait spaces and for future derivations of the trade-off from cellular allocation principles without implying that the particular power law used here is unique.

### 10.2. Eco-Evolutionary Regime Dependence

Rapid evolution can occur on ecological timescales [54]. Laboratory experiments have shown that prey evolution can reshape predator–prey dynamics [53], and co-evolution of bacteria and phages has been observed directly in continuous chemostat culture [63]. These systems motivate the question of how the thermodynamic trajectory changes when ecological and evolutionary timescales become comparable.

The frozen-time Routh–Hurwitz calculation supplies a local spectral boundary for this regime. Equation (40) adds a biologically informative restriction because the threshold requires $d_{m}>0$; this particular instability mechanism is therefore absent from the ideal chemostat, in which biomass loss occurs entirely through washout. In the unbounded model, the admissible trait interval closes as $\bar{k}$ increases. A hard cap on the mean trait can keep the interval open, whereas a smooth interior trade-off optimum suppresses the first-order selection gradient and stabilises the corresponding local feedback. These results specify where the instantaneous linearisation predicts local amplification, but they do not establish a persistent periodic orbit, which would require a separate bifurcation analysis of a fully specified nonlinear evolutionary model.

Taken together, the quasi-static, bounded-trait, and fast-regime results motivate what we call **Bounded Dissipative Adaptation**—a descriptive, regime-dependent picture of how selection, resource balance, and biochemical constraints jointly shape metabolic entropy production, rather than a new universal thermodynamic principle. Selection can increase metabolic entropy production by favouring phenotypes that exploit the limiting resource more effectively. Open-system mass balance sets an outer physical ceiling, while biochemical and proteomic constraints define tighter biologically attainable levels. When ecological and evolutionary timescales become comparable, the dynamical route itself can change, and the quasi-static monotonic description need no longer apply.

The framework therefore identifies both the scope and the limits of the thermodynamic tendency derived here. Within the specified quasi-static regime, natural selection has a monotonic thermodynamic signature, mass balance determines an ideal physical ceiling, and biological constraints can lower the attainable level or alter the route by which it is approached. Some natural extensions would be to include strain-dependent catabolic energetics and yields; derive proteomic trade-offs endogenously; retain fully saturating Michaelis–Menten fitness in the mutation–selection dynamics; and test the predicted relationships among resource drawdown, phenotype distributions, and metabolic dissipation in controlled continuous cultures.

## Data Availability

This work is entirely theoretical; no new experimental data were generated. All mathematical derivations and analytical proofs are contained within the manuscript.

## Appendix A. Notation

**Table A1:** Complete notation for all variables and parameters used in the manuscript, grouped by the description in which they appear. The manuscript moves between discrete strain-level, continuous trait-density, CRN/thermodynamic, trade-off, and fast-evolution descriptions. In the table, Varφ denotes the discrete uptake-rate variance, whereas Var(k,t) denotes continuous phenotypic variance. The uptake curvature is written $M_{U}$ when the full Michaelis–Menten dependence has already been evaluated at $C^{*}$ and as $M_{u}$ for the corresponding per-nutrient curvature, with $M_{U}=C^{*}M_{u}$.

Symbol	SI Units	Description
*Core ecological variables (discrete descriptions, Section 2, Section 3, Section 4, Section 5 and Section 6)*		
*J*	g L^−1^ s^−1^	Constant nutrient influx rate
C(t)	g L^−1^	Nutrient concentration at time *t*
$x_{i}\left(t\right)$	g L^−1^	Biomass density of strain *i*
X(t)	g L^−1^	Total biomass: $X=\sum_{i}x_{i}$
$p_{i}\left(t\right)$	dimensionless	Relative frequency: $p_{i}=x_{i}/X$
γ	$g_{\mathrm{biom}}$ $g_{\mathrm{nutr}}^{-1}$	Biomass yield coefficient
*d*	s^−1^	Mortality/washout rate
κ	s^−1^	Physical removal rate of nutrient
$v_{\max,i}$	s^−1^	Maximum specific uptake rate of strain *i*
$K_{m,i}$	g L^−1^	Michaelis constant of strain *i*
$\varphi_{i}\left(C\right)$	s^−1^	Specific uptake rate (Equation (1))
$\bar{\varphi }\left(C,t\right)$	s^−1^	Population-mean specific uptake rate
$\bar{v}\left(t\right)$, $\bar{K}\left(t\right)$	(mixed)	Frequency-weighted effective MM parameters (Section 4)
$C^{*}$, $X^{*}$	g L^−1^	Ecological equilibrium values
Varφ(C,t)	s^−2^	*Discrete* uptake-rate variance: $\sum_{i}p_{i}{\left[\varphi_{i}-\bar{\varphi }\right]}^{2}$; see note above
$M_{0}$	—	Quasi-static ecological manifold ($\bar{\varphi }\left(C^{*}\left(p\right),p\right)=d/\gamma$)
*Thermodynamic variables (Section 2.2, Section 2.3, Section 2.4 amd Section 3, Appendix B)*		
ΔS	J K^−1^ g^−1^	Specific entropy per unit nutrient metabolised
$\sigma \left(t\right)\equiv \sigma_{\mathrm{met}}\left(t\right)$	J K^−1^ L^−1^ s^−1^	Metabolic (internal) volumetric entropy production rate
$S_{\mathrm{sys}}$, ${\dot{S}}_{\mathrm{in}/\mathrm{out}}$, ${\dot{Q}}_{\mathrm{in}}$, $T_{\mathrm{env}}$	(mixed)	System entropy balance terms (Section 2)
$E_{i}$, $EC_{i}$	—	Mesoscopic enzyme/receptor states (Appendix B)
$k_{1,i},k_{-1,i},k_{2,i},k_{-2,i}$	(units of rate constants)	CRN forward/reverse rate constants
$\mu_{C}^{0},\mu_{E_{i}}^{0},\mu_{EC_{i}}^{0},\mu_{X_{i}}^{0}$	J mol^−1^	Standard-state molar chemical potentials
$A_{i}^{\mathrm{mol}}$	J mol^−1^	Molar thermodynamic affinity of the uptake cycle
*c*, $x_{i}^{\left(a\right)}$, $K_{i}$	mol L^−1^	Molar nutrient concentration, activity-equivalent biomass, CRN concentration scale
$e_{i}$, $M_{C}$	(mixed)	Effective receptor abundance per unit biomass; molar mass of nutrient
*Continuous description (trait density, Section 7 and Section 8)*		
ρ(k,t)	(L g^−1^ s^−1^)^−1^	Density over phenotype space (∫ρdk=1)
*k*	L g^−1^ s^−1^	Continuous phenotypic variable ($=v_{\max}/K_{m}$)
$\bar{k}\left(t\right)$	L g^−1^ s^−1^	Mean phenotype: ∫kρdk
Var(k,t), or bare Var	(L g^−1^ s^−1^)^2^	*Continuous* phenotypic variance: $\int {\left(k-\bar{k}\right)}^{2}\rho \;dk$; see note above
$D_{\mu }$	(L g^−1^ s^−1^)^2^ s^−1^	Mutational diffusion coefficient
$\mu_{3}\left(t\right)$, $\mu_{4}$	(L g^−1^ s^−1^)^3,4^	Third/fourth central moments of ρ(k,t)
*Trade-off model (Section 6.3)*		
$A_{\mathrm{tr}}$, β, *q*	(mixed)	Trade-off amplitude, exponent, and q=1/(1−β)
$k^{†}\left(C\right)$	L g^−1^ s^−1^	Interior fitness-optimal trait value at nutrient level *C*
U(k,C)	s^−1^	Full (saturating) uptake rate under the trade-off
$M_{U}$, $M_{u}$	$M_{U}$: g^2^ s L^−2^; $M_{u}$: g s L^−1^	Full-rate ($M_{U}$, Section 6.3) and per-nutrient ($M_{u}$, Section 7.2) stabilising curvature; $M_{U}=C^{*}M_{u}$
${\mathrm{Var}}^{†}$	(L g^−1^ s^−1^)^2^	Stationary phenotypic variance under stabilising selection
*Fast-evolution/stability variables (Section 9)*		
δ, $\tau_{\mathrm{eco}}$, $\tau_{\mathrm{evo}}$	(mixed)	Timescale-separation parameter and the two timescales it compares
*V*	(L g^−1^ s^−1^)^2^	Frozen instantaneous value of Var, held fixed in the spectral criterion
$J_{\mathrm{fr}}$	(mixed)	Frozen $\left(C,X,\bar{k}\right)$ Jacobian matrix (Equation (38))
$a_{2},a_{1},a_{0}$	(mixed)	Characteristic-polynomial coefficients of $J_{\mathrm{fr}}$
$V_{c}$	(L g^−1^ s^−1^)^2^	Critical frozen variance at the Routh–Hurwitz margin

**Notation note:** Superscript ^∗^ denotes an ecological-equilibrium value; superscript ^†^ denotes an optimum/stationary value associated with the trade-off or stabilising-selection model, as specified in the Description column.

## Appendix B. Chemical Reaction Network Derivation of the Michaelis–Menten Flux

### Appendix B.1. Microscopic Reaction Network and Local Detailed Balance

Nutrient uptake by strain *i* proceeds via a two-step reversible reaction with a surface enzyme/receptor $E_{i}$:

(A1) $$C+E_{i}\;\underset{k_{-1,i}}{\overset{k_{1,i}}{⇌}}\;EC_{i}\;\underset{k_{-2,i}}{\overset{k_{2,i}}{⇌}}\;\mathrm{biomass}+E_{i}.$$

Using molar chemical potentials, Local Detailed Balance (LDB) constrains the rate constants:

(A2) $$\frac{k_{1,i}}{k_{-1,i}}=\exp\;\left[\frac{-\mu_{EC_{i}}^{0}+\mu_{C}^{0}+\mu_{E_{i}}^{0}}{RT}\right],\;\frac{k_{2,i}}{k_{-2,i}}=\exp\;\left[\frac{-\mu_{X_{i}}^{0}-\mu_{E_{i}}^{0}+\mu_{EC_{i}}^{0}}{RT}\right].$$

### Appendix B.2. Thermodynamic Affinity, Net Flux, and the Biomass Normalisation

The overall coarse-grained cycle affinity, expressed on a molar basis, is $A_{i}^{\mathrm{mol}}=\mu_{C}-\mu_{X_{i}}$. Under ideal–dilute, activity-normalised conditions, this can be written as

(A3) $$A_{i}^{\mathrm{mol}}=RT\ln\;\left(\frac{k_{1,i}\;k_{2,i}\;c}{k_{-1,i}\;k_{-2,i}\;x_{i}^{\left(a\right)}}\right),$$

with activities defined on the same reference basis as the CRN rate constants. To keep the thermodynamic and ecological units explicit, let $c\equiv C/M_{C}$ denote the molar concentration corresponding to the ecological mass concentration *C*, and let $x_{i}^{\left(a\right)}\equiv \chi_{i}\;x_{i}$ denote the activity-equivalent concentration associated with the population-level biomass variable, where $\chi_{i}$ absorbs the coarse-grained conversion between biomass mass density and the effective product activity. Then *c*, $K_{i}$, and $x_{i}^{\left(a\right)}$ have the same concentration units in the reduced CRN rate law. The ecological variables *C* and $x_{i}$ remain mass concentrations throughout; the molar reaction flux is converted to nutrient-mass flux through $J_{\mathrm{met},i}^{n}=M_{C}\;J_{\mathrm{net},i}^{\mathrm{mol}}$. The corresponding mass-specific affinity is $A_{i}=A_{i}^{\mathrm{mol}}/M_{C}$, where $M_{C}$ is the molar mass of the nutrient. Under the quasi-steady-state approximation for $EC_{i}$ with total receptor conservation $E_{0,i}=\left[E_{i}\right]+\left[EC_{i}\right]$, the net molar flux is

(A4) $$J_{\mathrm{net},i}^{\mathrm{mol}}\left(c,x_{i}^{\left(a\right)}\right)=\frac{k_{2,i}\;E_{0,i}\;c\;\left(1-e^{-A_{i}^{\mathrm{mol}}/RT}\right)}{K_{i}+c+\frac{k_{-2,i}}{k_{1,i}}\;x_{i}^{\left(a\right)}},\;K_{i}\equiv \frac{k_{-1,i}+k_{2,i}}{k_{1,i}}.$$

If one writes $E_{0,i}=e_{i}\;x_{i}$, where $e_{i}$ is an effective receptor abundance per unit biomass, the specific mass uptake flux is

(A5) $$\varphi_{i}\left(C,x_{i}\right)\equiv \frac{J_{\mathrm{met},i}^{n}}{x_{i}}=\frac{\left(M_{C}\;k_{2,i}\;e_{i}\right)\;c\;\left(1-e^{-A_{i}^{\mathrm{mol}}/RT}\right)}{K_{i}+c+\frac{k_{-2,i}}{k_{1,i}}\;x_{i}^{\left(a\right)}}.$$

Writing $e_{i}$ in mol of effective receptor per gram of biomass gives $v_{\max,i}=M_{C}\;k_{2,i}\;e_{i}$ in s^−1^. The ecological half-saturation constant and the CRN concentration scale are related by $K_{m,i}=M_{C}\;K_{i}$. Hence, Equation (A5) has the same units as the ecological specific rate $\varphi_{i}\left(C\right)$ of Equation (1).

The assumption $E_{0,i}=e_{i}\;x_{i}$ with $e_{i}$ constant treats the effective receptor abundance per unit biomass as a fixed, strain-specific parameter. The evolutionary dynamics therefore focus on enzyme kinetic parameters, while regulatory expression is reserved for future extensions. Living cells operate far from thermodynamic equilibrium; in the biologically relevant forward-flux regime, $A_{i}^{\mathrm{mol}}\gg RT$ and $\left(k_{-2,i}/k_{1,i}\right)\;x_{i}^{\left(a\right)}\ll K_{i}+c$, and so the reversible flux above reduces to Equation (11) of the main text.

## Appendix C. Proofs of the Fisher-Type Lemma, the Monotonicity Theorem, and the Exact Moment Equations

### Appendix C.1. Proof of Lemma 1

**Proof of Lemma 1.** Differentiating $\bar{\varphi }=\sum_{i}p_{i}\;\varphi_{i}\left(C\right)$ by the product rule gives Equation (21). Substitute ${\dot{p}}_{i}=\gamma \;p_{i}\;\left(\varphi_{i}-\bar{\varphi }\right)$ into the first term:

$$\sum_{i}{\dot{p}}_{i}\;\varphi_{i}\left(C\right)=\gamma \sum_{i}p_{i}\;\left[\varphi_{i}-\bar{\varphi }\right]\;\varphi_{i}=\gamma \;\left(\sum_{i}p_{i}\;\varphi_{i}^{2}-{\bar{\varphi }}^{2}\right)=\gamma \;\mathrm{Var}\;\varphi \left(C,t\right).$$

Restricting to $M_{0}$ completes the proof. □

### Appendix C.2. Proof of Theorem 1

**Proof of Theorem 1.** On $M_{0}$, $\bar{\varphi }\left(C^{*}\left(t\right),p\left(t\right)\right)=d/\gamma$ for all *t*. Differentiate

$$\alpha \;{\dot{C}}^{*}+\gamma \sum_{i}p_{i}\;{\left[\varphi_{i}\left(C^{*}\right)-\bar{\varphi }\right]}^{2}=0\Rightarrow {\dot{C}}^{*}=-\frac{\gamma }{\alpha }\;\mathrm{Var}\;\varphi \left(C^{*},t\right).$$

Differentiate $\sigma^{*}=\Delta S\;\left(J-\kappa C^{*}\right)$:

$$\frac{d\sigma^{*}}{dt}=-\Delta S\;\kappa \;{\dot{C}}^{*}=\frac{\Delta S\;\kappa \;\gamma }{\alpha }\;\mathrm{Var}\;\varphi \left(C^{*},t\right)\geq 0.\;$$

□

### Appendix C.3. Proof of Theorem 2

**Proof of Theorem 2.** Multiply Equation (31) by *k* and integrate:

$$\frac{d\bar{k}}{dt}=\gamma C\int k\;\left(k-\bar{k}\right)\;\rho \;dk+D_{\mu }\int k\;\frac{\partial^{2}\rho }{\partial k^{2}}\;dk=\gamma C\;\mathrm{Var}\left(k\right).$$

Multiply by $k^{2}$:

$$\frac{d\langle k^{2}\rangle }{dt}=\gamma C\int k^{2}\;\left(k-\bar{k}\right)\;\rho \;dk+D_{\mu }\int k^{2}\;\frac{\partial^{2}\rho }{\partial k^{2}}\;dk=\gamma C\;\left(\mu_{3}+2\bar{k}\;\mathrm{Var}\right)+2D_{\mu }.$$

Use $\mathrm{Var}=\langle k^{2}\rangle -{\bar{k}}^{2}$:

$$\frac{d\;\mathrm{Var}}{dt}=\frac{d\langle k^{2}\rangle }{dt}-2\bar{k}\;\frac{d\bar{k}}{dt}=\gamma C\;\mu_{3}+2D_{\mu }.\;$$

□

### Appendix C.4. Proof of Theorem 3

**Proof of Theorem 3.** From Equation (34): $\mathrm{Var}\left(t\right)={\mathrm{Var}}_{0}+2D_{\mu }\;t$. Substitute into the $\bar{k}$ equation:

$$\bar{k}\;d\bar{k}=d\;\left({\mathrm{Var}}_{0}+2D_{\mu }\;t\right)\;dt\Rightarrow \frac{{\bar{k}}^{2}}{2}=d\;{\mathrm{Var}}_{0}\;t+d\;D_{\mu }\;t^{2}+\mathrm{const}.$$

Hence, $\bar{k}\left(t\right)=\sqrt{{\bar{k}}_{0}^{2}+2d\;{\mathrm{Var}}_{0}\;t+2d\;D_{\mu }\;t^{2}}$. Since $\bar{k}\left(t\right)\to \infty$:$C^{*}\left(t\right)=d/\left(\gamma \bar{k}\left(t\right)\right)\to 0$ and $\sigma^{*}\left(t\right)=\Delta S\;\left(J-\kappa d/\left(\gamma \bar{k}\left(t\right)\right)\right)\to \Delta S\cdot J$. □

## Appendix D. Full Jacobian Algebra for the Frozen Spectral Criterion

Expanding $\det(J_{\mathrm{fr}}-\lambda I)=0$ for the matrix of Equation (38) by cofactor expansion along the second column (whose only zero entry is in the third row) gives

$$\det\left(J_{\mathrm{fr}}-\lambda I\right)=\left(-\bar{k}\;C^{*}\right)\left[\gamma \;\bar{k}\;X^{*}\;\lambda +\gamma^{2}\;C^{*}\;X^{*}\;V\right]+\left(-\lambda \right)\left[\lambda^{2}+\left(\kappa +\bar{k}\;X^{*}\right)\;\lambda +\gamma \;C^{*}\;X^{*}\;V\right].$$

Collect powers of λ:

$$\det\left(J_{\mathrm{fr}}-\lambda I\right)=-\lambda^{3}-\left(\kappa +\bar{k}\;X^{*}\right)\;\lambda^{2}-\gamma \;C^{*}\;X^{*}\;\left({\bar{k}}^{2}+V\right)\;\lambda -\gamma^{2}\;\bar{k}\;{\left(C^{*}\right)}^{2}\;X^{*}\;V,$$

and so $\det(J_{\mathrm{fr}}-\lambda I)=0$ is equivalent to the characteristic polynomial

(A6) $$\lambda^{3}+a_{2}\;\lambda^{2}+a_{1}\;\lambda +a_{0}=0,$$

with

(A7) $$a_{2}=\kappa +\bar{k}\;X^{*},\;a_{1}=\gamma \;C^{*}\;X^{*}\;\left({\bar{k}}^{2}+V\right),\;a_{0}=\gamma^{2}\;\bar{k}\;{\left(C^{*}\right)}^{2}\;X^{*}\;V.$$

Equivalently, $a_{2}=-\mathrm{Tr}\left(J_{\mathrm{fr}}\right)$, $a_{1}$ is the sum of the three 2×2 principal minors of $J_{\mathrm{fr}}$, and $a_{0}=-\det\left(J_{\mathrm{fr}}\right)$, as required for the characteristic polynomial of a 3×3 matrix.

The Routh–Hurwitz stability condition for a cubic $\lambda^{3}+a_{2}\lambda^{2}+a_{1}\lambda +a_{0}$ is $a_{2}>0$, $a_{0}>0$, and $a_{2}\;a_{1}>a_{0}$. Since $a_{2}>0$ and $a_{0}>0$ in all cases (both are sums or products of positive quantities), stability is controlled entirely by the sign of the margin

(A8) $$a_{2}\;a_{1}-a_{0}=\gamma \;C^{*}\;X^{*}\;\left[\left(\kappa +\bar{k}\;X^{*}-d\right)\;V+\left(\kappa +\bar{k}\;X^{*}\right)\;{\bar{k}}^{2}\right],$$

obtained by substituting $\gamma \bar{k}C^{*}=d$ into $a_{2}a_{1}-a_{0}$ and collecting terms in *V*. Setting this margin to zero and solving for *V* gives the threshold in Equation (39).

## References

[1] Schrödinger E. (1944). What Is Life?.

[2] Martyushev L.M., Seleznev V.D. (2006). Maximum entropy production principle in physics, chemistry and biology. Phys. Rep..

[3] Schneider E.D., Kay J.J. (1994). Life as a manifestation of the second law of thermodynamics. Math. Comput. Model..

[4] Michaelian K. (2011). Thermodynamic dissipation theory for the origin of life. Earth Syst. Dyn..

[5] Dewar R. (2003). Information theory explanation of the fluctuation theorem, maximum entropy production and self-organized criticality. J. Phys. A.

[6] Kleidon A., Lorenz R.D. (2005). Non-Equilibrium Thermodynamics and the Production of Entropy.

[7] Volk T., Pauluis O. (2010). It is not the entropy you produce, rather, how you produce it. Philos. Trans. R. Soc. B.

[8] Meysman F.J.R., Bruers S. (2010). Ecosystem functioning and maximum entropy production. Philos. Trans. R. Soc. B.

[9] Bruers S. (2007). A discussion on maximum entropy production and information theory. J. Phys. A.

[10] Seifert U. (2012). Stochastic thermodynamics, fluctuation theorems and molecular machines. Rep. Prog. Phys..

[11] Rao R., Esposito M. (2016). Nonequilibrium thermodynamics of chemical reaction networks. Phys. Rev. X.

[12] Qian H. (2007). Phosphorylation energy hypothesis: Open chemical systems and their biological functions. Annu. Rev. Phys. Chem..

[13] Battle C., Broedersz C.P., Fakhri N., Geyer V.F., Howard J., Schmidt C.F., MacKintosh F.C. (2016). Broken detailed balance at mesoscopic scales in active biological systems. Science.

[14] Hill T.L. (1977). Free Energy Transduction in Biology.

[15] Hill T.L. (1989). Free Energy Transduction and Biochemical Cycle Kinetics.

[16] Schnakenberg J. (1976). Network theory of microscopic and macroscopic behavior of master equation systems. Rev. Mod. Phys..

[17] Westerhoff H.V., van Dam K. (1987). Thermodynamics and Control of Biological Free-Energy Transduction.

[18] Qian H., Beard D.A. (2005). Thermodynamics of stoichiometric biochemical networks in living systems far from equilibrium. Biophys. Chem..

[19] Qian H. (2006). Open-system nonequilibrium steady state: Statistical thermodynamics, fluctuations, and chemical oscillations. J. Phys. Chem. B.

[20] Beard D.A., Qian H. (2008). Chemical Biophysics: Quantitative Analysis of Cellular Systems.

[21] Ge H., Qian H. (2010). Physical origins of entropy production, free energy dissipation, and their mathematical representations. Phys. Rev. E.

[22] Andrieux D., Gaspard P. (2007). Fluctuation theorem for currents and Schnakenberg network theory. J. Stat. Phys..

[23] Avanzini F., Freitas N., Esposito M. (2023). Circuit theory for chemical reaction networks. Phys. Rev. X.

[24] Wachtel A., Rao R., Esposito M. (2018). Thermodynamically consistent coarse graining of biocatalysts beyond Michaelis–Menten. New J. Phys..

[25] Dobovišek A., Županovič P., Brumen M., Bonačič-Lošić Ž., Kučić D., Juretić D. (2011). Enzyme kinetics and the maximum entropy production principle. Biophys. Chem..

[26] Dobovišek A., Markovič R., Brumen M., Fajmut A. (2018). The maximum entropy production and maximum Shannon information entropy in enzyme kinetics. Phys. A.

[27] Dobovišek A., Vitas M., Blaževič T., Markovič R., Marhl M., Fajmut A. (2023). Self-Organization of Enzyme-Catalyzed Reactions Studied by the Maximum Entropy Production Principle. Int. J. Mol. Sci..

[28] Unrean P., Srienc F. (2011). Metabolic networks evolve towards states of maximum entropy production. Metab. Eng..

[29] Stucki J.W. (1980). The optimal efficiency and the economic degrees of coupling of oxidative phosphorylation. Eur. J. Biochem..

[30] Caplan S.R., Essig A. (1983). Bioenergetics and Linear Nonequilibrium Thermodynamics: The Steady State.

[31] Heinrich R., Schuster S. (1996). The Regulation of Cellular Systems.

[32] Fell D.A. (1997). Understanding the Control of Metabolism.

[33] Kümmel A., Panke S., Heinemann M. (2006). Systematic assignment of thermodynamic constraints in metabolic network models. BMC Bioinform..

[34] Henry C.S., Broadbelt L.J., Hatzimanikakis V. (2007). Thermodynamics-based metabolic flux analysis. Biophys. J..

[35] Molenaar D., van Berlo R., de Ridder D., Teusink B. (2009). Shifts in growth strategies reflect tradeoffs in cellular economics. Mol. Syst. Biol..

[36] von Stockar U., Maskow T., Liu J., Marison I.W., Patiño R. (2006). Thermodynamics of microbial growth and metabolism: An analysis of the current situation. J. Biotechnol..

[37] von Stockar U. (2010). Biothermodynamics of live cells: A tool for biotechnology and biochemical engineering. J. Non-Equilib. Thermodyn..

[38] Baez J.C. (2021). The fundamental theorem of natural selection. Entropy.

[39] Frank S.A. (2012). Natural selection. V. How to read the fundamental equations of evolutionary change in terms of information theory. J. Evol. Biol..

[40] Frank S.A. (2017). Universal expressions of population change by the Price equation: Natural selection, information, and maximum entropy production. Ecol. Evol..

[41] England J.L. (2013). Statistical physics of self-replication. J. Chem. Phys..

[42] England J.L. (2015). Dissipative adaptation in driven self-assembly. Nat. Nanotechnol..

[43] Perunov N., Marsland R.A., England J.L. (2016). Statistical physics of adaptation. Phys. Rev. X.

[44] Kolchinsky A. (2025). Thermodynamics of Darwinian selection in molecular replicators. Philos. Trans. R. Soc. B.

[45] Piñero J., Sowinski D.R., Ghoshal G., Frank A., Kolchinsky A. (2026). Information bounds production in replicator systems. Commun. Phys..

[46] Tabanera-Bravo J., Godec A. (2025). Multiple Pareto-optimal solutions of the dissipation–adaptation trade-off. Phys. Rev. Res..

[47] Juretić D., Bruvo Mađarić B. (2026). Enzyme Catalytic Parameters and Evolution Across the Dissipation Plane. Int. J. Mol. Sci..

[48] Smith H.L., Waltman P. (1995). The Theory of the Chemostat: Dynamics of Microbial Competition.

[49] Lenski R.E., Rose M.R., Simpson S.C., Tadler S.C. (1991). Long-term experimental evolution in *Escherichia coli*. Am. Nat..

[50] Sawada Y., Daigaku Y., Toma K. (2025). Maximum entropy production principle of thermodynamics for the birth and evolution of life. Entropy.

[51] Lineweaver C.H. (2025). Beyond the second law: Darwinian evolution as a tendency for entropy production to increase. Entropy.

[52] de Groot S.R., Mazur P. (1962). Non-Equilibrium Thermodynamics.

[53] Yoshida T., Jones L.E., Ellner S.P., Fussmann G.F., Hairston N.G. (2003). Rapid evolution drives ecological dynamics in a predator–prey system. Nature.

[54] Hairston N.G., Ellner S.P., Geber M.A., Yoshida T., Fox J.A. (2005). Rapid evolution and the convergence of ecological and evolutionary time. Ecol. Lett..

[55] Camacho Mateu J., Sireci M., Muñoz M.A. (2021). Phenotypic-dependent variability and the emergence of tolerance in bacterial populations. PLoS Comput. Biol..

[56] Reguera D., Rubí J.M., Vilar J.M.G. (2005). The mesoscopic dynamics of thermodynamic systems. J. Phys. Chem. B.

[57] Hofbauer J., Sigmund K. (1998). Evolutionary Games and Population Dynamics.

[58] Qian H. (2014). Fitness and entropy production in a cell population dynamics with epigenetic phenotype switching. Quant. Biol..

[59] Arango-Restrepo A., Barragán D., Rubí J.M. (2021). A criterion for the formation of nonequilibrium self-assembled structures. J. Phys. Chem. B.

[60] Lande R. (1976). Natural selection and random genetic drift in phenotypic evolution. Evolution.

[61] Turelli M. (1984). Heritable genetic variation via mutation–selection balance. Theor. Popul. Biol..

[62] Crow J.F., Kimura M. (1970). An Introduction to Population Genetics Theory.

[63] Mizoguchi K., Morita M., Fischer C.R., Yoichi M., Tanji Y., Unno H. (2003). Coevolution of Bacteriophage PP01 and *Escherichia coli* O157:H7 in Continuous Culture. Appl. Environ. Microbiol..

[64] Lotka A.J. (1922). Contribution to the energetics of evolution. Proc. Natl. Acad. Sci. USA.

[65] Ulanowicz R.E., Hannon B.M. (1987). Life and the production of entropy. Proc. R. Soc. Lond. B.

[66] Bar-Even A., Noor E., Savir Y., Liebermeister W., Davidi D., Tawfik D.S., Milo R. (2011). The moderately efficient enzyme. Biochemistry.

[67] Tawfik D.S. (2014). Accuracy-rate tradeoffs: How do enzymes meet demands of selectivity and catalytic efficiency?. Curr. Opin. Chem. Biol..

[68] Scott M., Gunderson C.W., Mateescu E.M., Zhang Z., Hwa T. (2010). Interdependence of cell growth and gene expression: Origins and consequences. Science.

[69] Dekel E., Alon U. (2005). Optimality and evolutionary tuning of the expression level of a protein. Nature.

[70] Peebo K., Valgepea K., Maser A., Nahku R., Adamberg K., Vilu R. (2015). Proteome reallocation in *Escherichia coli* with increasing specific growth rate. Mol. BioSyst..

[71] Schmidt A., Kochanowski K., Vedelaar S., Ahrné E., Volkmer B., Callipo L., Knoops K., Bauer M., Aebersold R., Heinemann M. (2016). The quantitative and condition-dependent *Escherichia coli* proteome. Nat. Biotechnol..

